# Identification of *RASSF1A* promoter hypermethylation as a biomarker for hepatocellular carcinoma

**DOI:** 10.1186/s12935-020-01638-5

**Published:** 2020-11-10

**Authors:** Gang Xu, Xiaoxiang Zhou, Jiali Xing, Yao Xiao, Bao Jin, Lejia Sun, Huayu Yang, Shunda Du, Haifeng Xu, Yilei Mao

**Affiliations:** Department of Liver Surgery, Peking Union Medical College (PUMC) Hospital, PUMC and Chinese Academy of Medical Sciences, Beijing, 100730 China

**Keywords:** *RASSF1A* promoter hypermethylation, Hepatocellular carcinoma, Biomarker, Overall survival, Diagnosis

## Abstract

**Background:**

RAS association domain family protein 1A (*RASSF1A*) promoter hypermethylation is suggested to be linked to hepatocellular carcinoma (HCC), but the results remained controversial.

**Methods:**

We evaluated how *RASSF1A* promoter hypermethylation affects HCC risk and its clinicopathological characteristics through meta-analysis. Data on DNA methylation in HCC and relevant clinical data were also collected based on The Cancer Genome Atlas (TCGA) database to investigate the prognostic role of *RASSF1A* promoter hypermethylation in HCC.

**Results:**

Forty-four articles involving 4777 individuals were enrolled in the pooled analyses. The *RASSF1A* promoter methylation rate was notably higher in the HCC cases than the non-tumor cases and healthy individuals, and was significantly related to hepatitis B virus (HBV) infection-positivity and large tumor size. Kaplan–Meier survival analysis revealed that HCC cases with *RASSF1A* promoter hypermethylation had worse outcomes. Receiver operating characteristic curves confirmed that *RASSF1A* promoter methylation may be a marker of HCC-related prognoses.

**Conclusions:**

*RASSF1A* promoter hypermethylation is a promising biomarker for the diagnosis of HCC from tissue and peripheral blood, and is an emerging therapeutic target against HCC.

## Introduction

Liver cancer (LC) is the sixth leading cause of cancer-related morbidity, and the fourth major cause of cancer-related death, worldwide. Approximately 841,000 newly diagnosed LC cases and 782,000 LC-related deaths are reported annually [1]. Hepatocellular carcinoma (HCC) is a major histological subtype of LC, accounting for 70% to 85% of all LC cases, globally [2]. While significant progress has been made in the diagnosis and treatment HCC, patients with the disease still have unsatisfactory prognoses [3]. Consequently, new clinical strategies are needed to improve the efficacy of HCC treatment, including the development of novel diagnostic and prognostic biomarkers.

Recent emerging evidence suggests that the accumulation of epigenetic and genetic alterations has a role in the different stages of liver carcinogenesis [4]. Besides, CpG island methylation within gene promoters, key epigenetic regulatory factors, has an important role in HCC initiation and development [5]. Promoter hypermethylation may result in the silencing of some tumor suppressors that regulate the cell signaling pathways in tumor tissues [6–8]. Among them, the RAS association domain family protein 1A (*RASSF1A*) is an important tumor suppressor associated with multiple biological functions, and its promoter is frequently blocked due to promoter hypermethylation in numerous malignant tumors, including HCC [4, 9]. The promoter hypermethylation of *RASSF1A* may have potential screening value, and may serve as an attractive early diagnostic and prognostic biomarker in HCC.

While a number of individual studies are being performed in patients with HCC, results on the association between *RASSF1A* promoter hypermethylation and HCC risk or its clinicopathological features remain controversial [10]. Although a study focusing on the diagnostic accuracy of the same has been conducted, only seven articles focusing on *RASSF1A* methylation in peripheral blood have been enrolled for analysis [11]. Moreover, it remains to be systemically investigated whether *RASSF1A* promoter hypermethylation is related to the clinicopathological features of HCC and the associated prognoses. Accordingly, we aimed to more comprehensively evaluate the role of *RASSF1A* promoter hypermethylation in HCC.

## Materials and methods

The study included two parts: meta-analysis and bioinformatic analysis. The meta-analysis was implemented in accordance with Preferred Reporting Items for Systematic Reviews and Meta-analyses guidelines [12]. The data from the TCGA are publicly available and open‐access; therefore, the local ethics committees did not need to approve the study because the current research follows the TCGA data access policies and publication guidelines.

### Retrieval and screening of eligible studies

Electronic databases, including Cochrane Library, Web of Science, EMbase, and Pubmed were searched for the identification of English-language articles from inception till April 30, 2020. The search strategy of (HCC OR hepatocellular carcinoma OR liver cancer) AND (hypermethylation OR methylation OR epigenetics) AND (*RASSF1A* or RASSF1 or Ras association domain family 1 A) was utilized for retrieval. In addition, the reference lists in relevant reviews and included studies were also checked manually for the avoidance of omission.

The study inclusion criteria were: (1) studies that reported on the relationship of the promoter methylation of *RASSF1A* with HCC or the associated clinicopathological characteristics in patients with HCC; (2) studies that investigated the *RASSF1A* promoter methylation levels in both tissues and blood; (3) case–control studies that regarded people with HCC as cases (as confirmed from HCC tissues and peripheral blood) and people without HCC as controls (as confirmed using adjacent noncancerous tissues, benign lesions, normal tissues, and serum); and (4) studies that reported the exact *RASSF1A* methylation frequency in both cases and controls. Meanwhile, studies not conforming to the inclusion criteria were excluded. For duplicate studies, the most complete report was selected.

### Extraction of data and evaluation of quality

Data were independently extracted by two reviewers following a pre-defined procedure. The data collected in this study included: author names, study design, year of publication, sample type, control sample, hypermethylated case number, hypomethylated case number, hypermethylated control number, hypomethylated control number, detection method and clinicopathologic parameters, such as age, sex, HBV infection, HCV infection, tumor number, tumor size, liver cirrhosis, AFP level, pathological grade, tumor differentiation and portal venous invasion.

Moreover, two reviewers independently evaluated the methodological quality of the enrolled studies according to the criteria stipulated in the NOS [13]. Each study was assigned a score ranging from 0 (poor quality) to 9 points (optimal quality) based on the selection, comparability and exposure of the cases and controls. Any disagreement between the two reviewers was settled by discussion.

### Meta-analysis

Stata 12.0 software (Stata Corporation, College Station, Texas, USA) and R software (version 3.4.4) were adopted for the statistical analyses and plotting. The ORs of the cases versus controls in each study were calculated by 2 × 2 tables. A classic half-integer continuity correction was applied in studies that reported zero events in the treatment or control arm. The log ORs were then aggregated to obtain combined results. Then, the results for the controls were pooled in the non-tumor group (including adjacent non-cancerous tissues, benign lesions, and serum from patients with benign disease) and the normal group (including liver tissues and serum from healthy donors). Moreover, the heterogeneity between two studies was evaluated through the I^2^ statistic and Chi square tests. The level of heterogeneity was deemed significant at I^2^ > 50% and *P* < 0.10 for Chi square tests. This study adopted the random-effects model for all analyses to obtain conservative results [14]. Subgroup analyses stratified by sample type, detection method and sample size were performed for the investigation of possible heterogeneity sources. Additionally, the covariate impacts on those integrated results as well as the heterogeneity across different studies were evaluated by meta-regression analysis.

In the sensitivity analysis, one study was eliminated at a time for the evaluation of its influence on the pooled analysis. Egger’s test and Begg’s test were utilized for the identification of publication bias [14, 15]. For both tests, *P* < 0.05 indicated significant publication bias. Furthermore, the presence of potential publication bias was adjusted by the “trim and fill” approach [16], which estimated the potential studies omitted and then re-calculated the integrated results with these hypothetical studies. *P *< 0.05 (two-tailed) indicated statistical significance.

### Extraction and analysis of TCGA data

Data on DNA methylation in HCC and corresponding clinical data were collected from the TCGA (Illumina Infinium Human Methylation 450 [HM450]) database including 485,577 probes. Then, the methylation levels of all the probes were determined by the β values. Empirical thresholds of 0.2 and 0.6 were adopted to distinguish between complete non-methylation, hypomethylation, and hypermethylation. Specifically, β ≤ 0.6 represented hypomethylation and β > 0.6 signified hypermethylation. Further, the Kaplan–Meier method was adopted for the construction of the OS and DFS curves for different *RASSF1A* methylation statuses, while the log-rank test was used for comparisons. In addition, this study also established time-dependent ROC curves, and determined the AUC values for the assessment of the predictive power of *RASSF1A* methylation status.

## Results

### Screening of studies

**Fig. 1:**
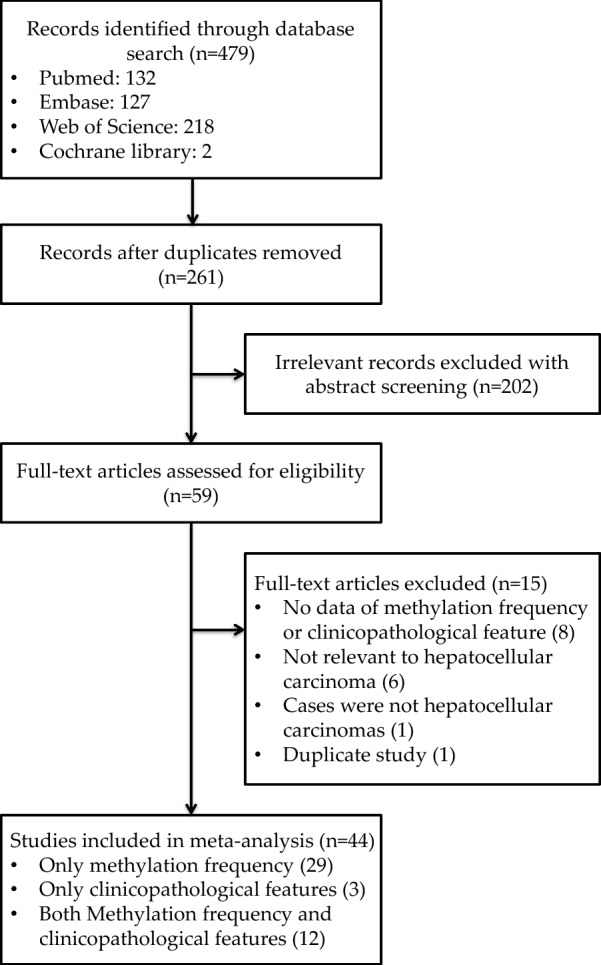
Flow diagram of the study selection process

Totally, 479 articles were identified through the original search strategy (Fig. 1). Of them, 218 duplicate articles were ruled out, and an additional 61 were eliminated due to the lack of relevance to this study after their abstracts were read. Later, the full-texts of the remaining 59 studies were carefully read; 44 satisfied our study inclusion criteria and were enrolled for analyses. In detail, 12 studies presented data on the *RASSF1A* promoter methylation rate within HCC and assessed the association of this methylation with clinicopathological characteristics. Besides, 29 studies only assessed the frequency of *RASSF1A* promoter methylation, while 3 only evaluated the clinicopathological characteristics.

### Characteristics of the enrolled articles

The features of the enrolled articles were shown in Table 1. Altogether, 44 case–control studies involving 4777 individuals published from 2002 to 2019 were enrolled in the analyses [10, 17–59]. Twenty-eight articles originated in Asia, consistent with the epidemiology of HCC. America produced the second highest number of enrolled papers (n = 8), followed by Africa (n = 5), while Italy and Germany published one article each. Three types of sample sources were predominantly investigated, including tissues (n = 31), peripheral blood (n = 11), and both tissues and peripheral blood (n = 2). In all our enrolled studies, HCC patients were regarded as ‘cases’; those without the disease were considered ‘controls’, and were assigned to the non-tumor group and normal group. Of those articles examining HCC risk, 11 used blood, 31 adopted tissues, and two examined both blood and tissues. Heterogeneous methods were adopted for the detection of the *RASSF1A* methylation status among the enrolled studies. The Newcastle-Ottawa scale (NOS) was adopted to assess the quality of the 41 articles that reported the *RASSF1A* methylation rates in the cases and controls, with scores ranging from 5 to 8, indicating a relatively high methodological quality (Additional file 1: Table S1). Another three studies that only reported on the disease’s clinicopathological characteristics were not eligible for NOS assessment, and were thus not evaluated.

**Table 1:** Main characteristics of the eligible studies

Study	Country	No. of patient	Case		Control		Control sample	Sample type	Detection method
			M	T	M	T			
Yu, 2002 [17]	China	33	29	29	24	29	Non-tumor	Tissue	MSP
					0	4	Normal		
Zhang, 2002 [18]	China	94	70	82	7	10	Non-tumor	Tissue	MSP
					0	12	Normal		
Lee, 2003 [19]	Korea	144	40	60	2	86	Non-tumor	Tissue	MSP
Undraga, 2003 [20]	USA	24	14	15	5	9	Non-tumor	Tissue	MSP
Zhong, 2003 [21]	China	23	23	23	7	23	Non-tumor	Tissue	BSP
Lehmann, 2005 [22]	Germany	131	40	41	53	83	Non-tumor	Tissue	qMSP
					16	28	Normal		
Park, 2005 [23]	Korea	27	12	27	0	27	Non-tumor	Tissue	MSP
Yeo, 2005 [24]	China	50	17	40	0	10	Normal	Blood	MSP
Calvisi, 2006 [25]	USA	80	52	80	32	80	Non-tumor	Tissue	MSP
Gioia, 2006 [26]	Italy	84	26	26	81	95	Non-tumor	Tissue	MSP
					11	13	Normal		
Oh, 2007 [27]	Korea	32	23	25	9	24	Non-tumor	Tissue	MSP
					2	7	Normal		
Zhang, 2007 [28]	China	100	35	50	3	50	Normal	Blood	MSP
Zhang, 2007 [29]	China	56	44	50	24	50	Non-tumor	Tissue	MSP
					0	6	Normal		
Chan, 2008 [30]	China	126	59	63	37	63	Non-tumor	Blood	MSRE-qPCR
Chang, 2008 [31]	China	70	12	19	4	17	Non-tumor	Tissue	MSP
			7	26	3	16		Blood	
Nishida, 2008 [32]	Japan	99	66	77	43	77	Non-tumor	Tissue	MSRE-qPCR
					10	22	Normal		
Su, 2008 [33]	China	50	50	50	50	50	Non-tumor	Tissue	MSP
Lou, 2009 [34]	China	86	57	60	54	81	Non-tumor	Tissue	MSP
					0	5	Normal		
Hu, 2010 [35]	China	45	31	35	18	35	Non-tumor	Tissue	MSP
			14	35	0	10	Normal	Blood	
Formeister, 2010 [36]	USA	49	43	43	31	45	Non-tumor	Tissue	MSP
Feng, 2010 [37]	USA	65	10	40	1	25	Normal	Tissue	Methylight
Saelee, 2010 [38]	Thailand	29	25	29	3	29	Normal	Tissue	MSP
Hua, 2011 [39]	China	55	30	47	9	47	Non-tumor	Tissue	MSRE-qPCR
			30	47	0	8	Normal		
Um, 2011 [10]	Korea	46	31	46	56	89	Non-tumor	Tissue	Methylight
Feng, 2012 [40]	China	103	82	103	40	103	Non-tumor	Tissue	MSP
Li, 2012 [41]	China	N.A.	N.A.	N.A.	N.A.	N.A.	N.A.	Tissue	MSP
Mohamed, 2012 [42]	Egypt	60	36	40	2	20	Normal	Blood	MSRE-qPCR
					25	40	Non-tumor		
Xu, 2013 [43]	China	87	72	87	66	87	Non-tumor	Tissue	Methylight
Zhang, 2013 [44]	China	123	48	48	47	83	Non-tumor	Tissue	MSP
					6	40	Normal		
Michailidi, 2014 [45]	USA	27	14	27	1	17	Non-tumor	Tissue	MSP
Zekri, 2014 [46]	Egypt	64	31	31	26	38	Non-tumor	Tissue	MSP
					0	13	Normal		
Feng, 2015 [47]	China	260	214	260	101	260	Non-tumor	Tissue	MSP
Huang, 2015 [48]	China	48	32	34	33	44	Non-tumor	Tissue	MSP
			16	31	2	10	Normal	Blood	
Lin, 2015 [49]	China	N.A.	N.A.	N.A.	N.A.	N.A.	N.A.	Tissue	Nested-MSP
Qu, 2015 [50]	China	55	31	35	26	35	Non-tumor	Tissue	MSP
					2	20	Normal		
Kanekiyo, 2015 [51]	Japan	N.A.	N.A.	N.A.	N.A.	N.A.	N.A.	Blood	qMSP
Villanueva, 2015 [52]	USA	231	82	221	10	10	Normal	Tissue	Pyrosequencing
Dong, 2015 [53]	China	584	122	190	26	234	Non-tumor	Blood	Methylight
			2	0	0	160	Normal		
Araújo, 2016 [54]	Brazil	24	15	17	2	7	Non-tumor	Tissue	Pyrosequencing
Liu, 2017 [55]	China	155	77	105	0	50	Normal	Blood	MSP
Mansour, 2017 [56]	Egypt	121	36	41	25	40	Non-tumor	Blood	MSRE-qPCR
					2	40	Normal		
Wu, 2017 [57]	USA	494	21	237	16	257	Normal	Blood	MSP
Pasha, 2019 [58]	Egypt	300	40	10	14	100	Non-tumor	Blood	MSP
				0	0	100	Normal		
Bendary, 2019 [59]	Egypt	443	108	188	22	202	Non-tumor	Blood	MSP
					10	53	Normal		

Abbreviations: M, methylated; T, total; MSP, methylation-specific polymerase chain reaction; BSP, bisulfite sequencing polymerase chain reaction; qMSP, quantitative methylation-specific polymerase chain reaction; MSRE-qPCR, methylation-sensitive restriction enzyme-quantitative polymerase chain reaction; N.A., not available; Y, yes; N, no

### Effect of *RASSF1A* promoter hypermethylation on HCC in the pooled analyses

#### Comparison of *RASSF1A* promoter hypermethylation between HCC and non-tumor groups

**Fig. 2:**
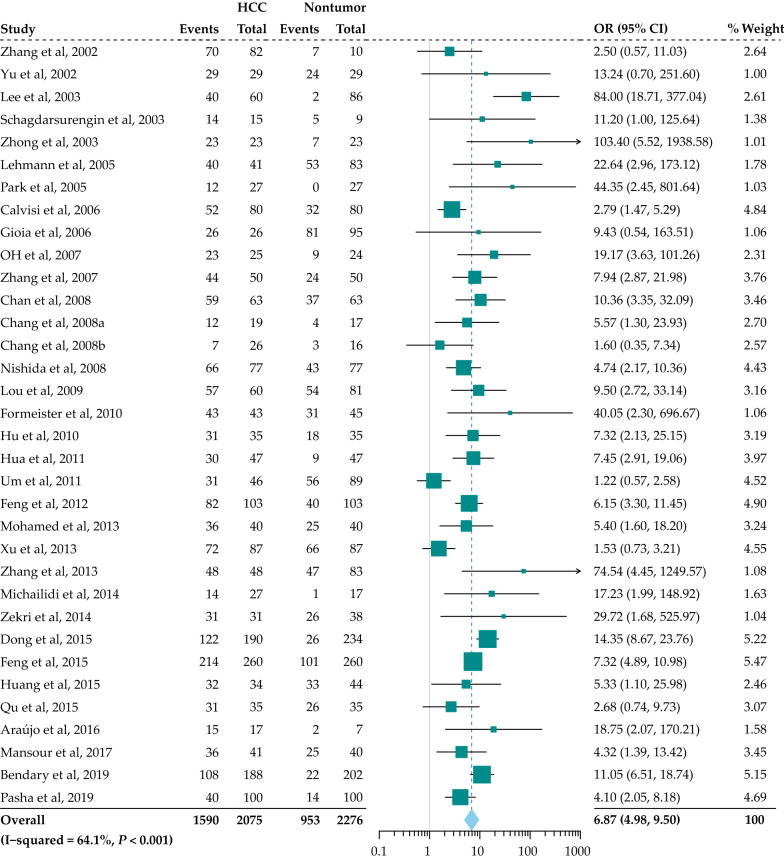
Forest plot of the correlation between *RASSF1A* promoter hypermethylation with HCC in non-tumor groups. OR, odds ratio; CI, confidence interval

Data from 34 studies including 2075 HCC patients and 2276 non-tumor controls underwent meta-analyses for the evaluation of the effect of *RASSF1A* promoter hypermethylation on HCC risk (Fig. 2). We found that the frequency of *RASSF1A* gene promoter hypermethylation was remarkably related to a high HCC risk in the overall comparison (odds ratio [OR] = 6.87, 95% confidence interval [CI] = 4.98–9.50, *P* < 0.001), and moderate heterogeneity was present (I^2^ = 64.1%, *P* = 0.000).

**Fig. 3:**
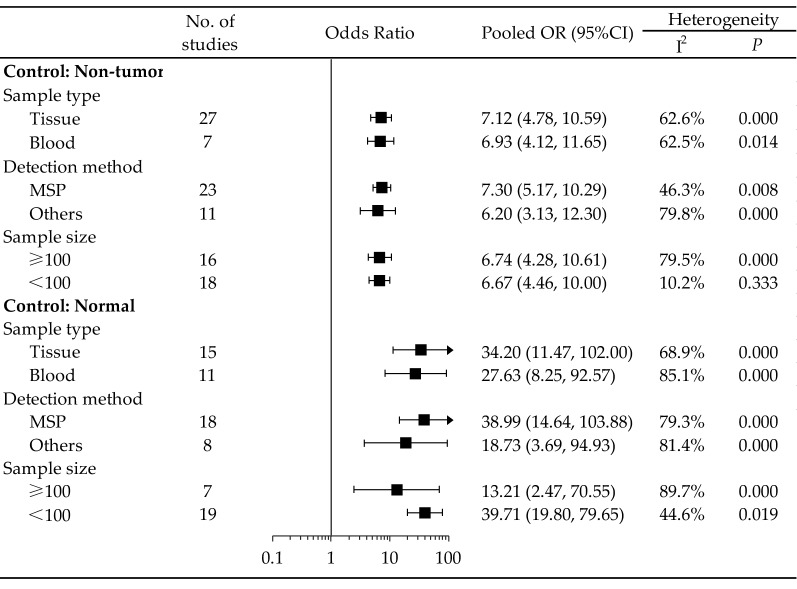
Forest plot of the subgroup analysis according to sample type (tissue and blood), detection method (MSP and others) and sample size (≥ 100 and < 100) for the correlation between *RASSF1A* promoter hypermethylation with HCC in non-tumor groups and normal groups. No., number; OR, odds ratio; CI, confidence interval

Further subgroup analyses stratified by sample type (blood and tissue), detection method (methylation-specific polymerase chain reaction [MSP] and others) and sample size (≥ 100 and < 100) were also performed to explore the possible heterogeneity sources across the various articles enrolled. Subgroup analyses stratified by sample type showed that *RASSF1A* gene promoter hypermethylation was significantly associated with HCC risk (blood: OR = 6.93, 95% CI = 4.12–11.65, *P* < 0.001; tissue: OR = 7.12, 95% CI = 4.78–10.59, *P*<0.001). In addition, in the subgroup analysis stratified by the detection method, *RASSF1A* gene promoter hypermethylation was evidently related to HCC risk (MSP: OR = 7.30, 95% CI = 5.17–10.29, *P* < 0.001; others: OR = 6.20, 95% CI = 3.13–12.30, P < 0.001). Similarly, the pooled results were consistent between the subgroups stratified by sample size (≥ 100: OR = 6.74, 95% CI = 4.28–10.61, *P*<0.001; < 100: OR = 6.67, 95% CI = 4.46–10.00, *P* < 0.001) (Fig. 3).

#### Comparison of *RASSF1A* promoter hypermethylation between HCC and normal groups

**Fig. 4:**
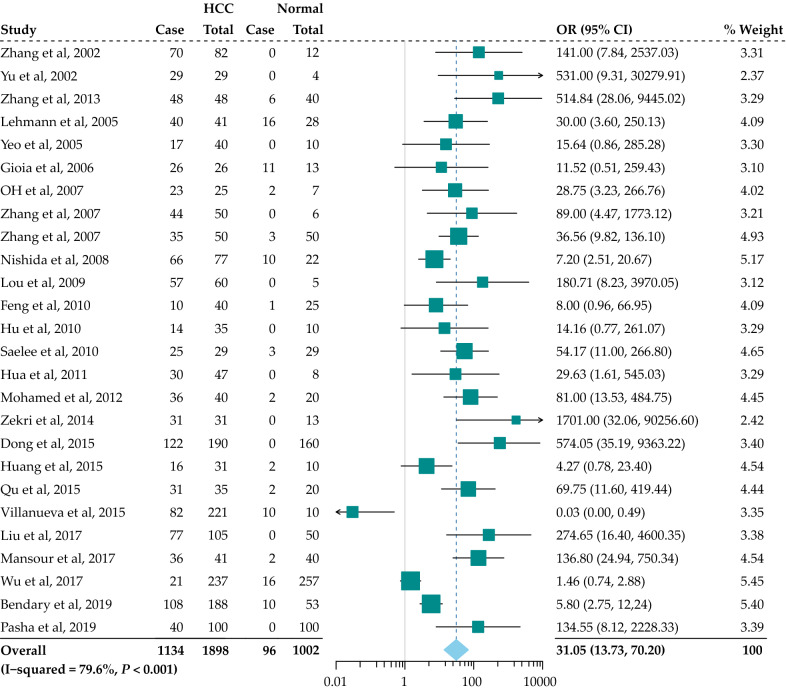
Forest plot of the correlation between *RASSF1A* promoter hypermethylation with HCC in normal groups. OR, odds ratio; CI, confidence interval

Totally, 26 studies enrolling 1898 HCC patients and 1002 normal controls were pooled for the assessment of how *RASSF1A* promoter hypermethylation affects HCC risk (Fig. 4). In the meta-analysis, the promoter methylation of *RASSF1A* was related to HCC risk in the cancer samples relative to the controls (OR = 31.05, 95% CI = 13.73–70.20, *P* < 0.001); in addition, a high heterogeneity level was detected across the various articles (I^2^ = 79.6%, *P* = 0.000).

Subgroup analyses revealed that the promoter methylation of *RASSF1A* was significantly correlated with the risk of HCC in all the subgroups stratified by sample type, detection method and sample size (Fig. 3).

### Relationship of the promoter hypermethylation of *RASSF1A* with the clinicopathological features

This study investigated a total of 11 characteristics from 15 studies that investigated the correlation of *RASSF1A* gene promoter hypermethylation with the clinicopathological features of HCC. The comprehensive data on the numerous clinicopathological features associated with HCC, and the association with the *RASSF1A* gene was presented in Table 2. As shown in the pooled analyses, *RASSF1A* promoter hypermethylation was remarkably related to tumor size (≥ 5 cm vs. < 5 cm, OR = 1.92, 95% CI = 1.07–3.42, *P* = 0.028) and hepatitis B virus (HBV) infection (positive vs. negative, OR = 1.50, 95% CI = 1.05–2.14, *P* = 0.026), but was not significantly associated with sex (male vs. female, OR = 1.36, 95% CI = 0.95–1.96, *P* = 0.094), age (≥ 50 vs. < 50, OR = 1.74, 95% CI = 0.82–3.69, *P* = 0.152), hepatitis C virus (HCV) infection (positive vs. negative, OR = 0.93, 95% CI = 0.20–4.26, *P* = 0.928), level of alpha fetoprotein (AFP) (≥ 20 μg/L vs. < 20 μg/L, OR = 1.25, 95% CI = 0.47–3.27, *P* = 0.657), tumor number (multiple vs. single, OR = 0.80, 95% CI = 0.47–1.36, *P* = 0.410), liver cirrhosis (presence vs. absence, OR = 1.06, 95% CI = 0.60–1.87, *P* = 0.834), histopathological stage (I + II vs. III + IV, OR = 1.84, 95% CI = 0.53–6.36, *P* = 0.338), tumor differentiation (poor vs. moderate or well, OR = 0.91, 95% CI = 0.41–2.02, *P* = 0.820) or portal venous invasion (presence vs. absence, OR = 0.61, 95% CI = 0.16–2.40, *P* = 0.481).

**Table 2:** Relationship of the promoter hypermethylation of *RASSF1A* with clinicopathological features of HCC

Parameters	No. of studies	Test for association			Test for heterogeneity (Random effect model)	
		OR	95% CI	*P*	I^2^ (%)	*P*
Age (≥ 50 vs.<50)	6	1.74	[0.82, 3.69]	0.152	0	0.622
Gender (male vs. female)	12	1.36	[0.95, 1.96]	0.094	0	0.894
HBV (positive vs. negative)	12	1.50	[1.05, 2.14]	*0.026*	0	0.720
HCV (positive vs. negative)	4	0.93	[0.20, 4.26]	0.928	0	0.707
Tumor number (multiple vs. single)	4	0.80	[0.47, 1.36]	0.410	13.9	0.323
Tumor size (≥ 5 cm vs. < 5 cm)	10	1.92	[1.07, 3.42]	*0.028*	38.8	0.100
Liver cirrhosis (Presence vs. Absence)	9	1.06	[0.60, 1.87]	0.834	55.8	0.021
AFP level (≥ 20 μg/L vs. < 20 μg/L)	6	1.25	[0.47, 3.27]	0.657	61.4	0.024
Histopathological grade (III/IV vs. I/II)	5	1.84	[0.53, 6.36]	0.338	81.3	< 0.001
Tumor differentiation (poor vs. moderate or well)	4	0.91	[0.41, 2.02]	0.820	0	0.778
Portal venous invasion (presence vs. absence)	4	0.61	[0.16, 2.40]	0.481	81.2	0.001

No., number; HBV, hepatitis B virus; HCV, hepatitis C virus; AFP, alpha-fetoprotein; OR, odds ratio; CI, confidence interval

### Meta-regression and sensitivity analyses

As for the results of the pooled meta-regression analysis on the correlation between the promoter hypermethylation of *RASSF1A* and HCC risk in both groups, a trend for sample type, detection method and sample size was demonstrated (Additional file 1: Table S2). Heterogeneity was detected in the pooled results; as a result, this study evaluated the contributions of diverse investigated features to heterogeneity. Nonetheless, there was no statistical significance (all *P* values > 0.05, Additional file 1: Table S2). The heterogeneity proportions in both groups ranged from − 9.70% to 8.14% (all *P* values > 0.05), with a high level of residual heterogeneity (τ^2^ range, 0.506–3.226). Owing to a lack of sufficient data in the enrolled articles, this study did not incorporate other factors that possibly contribute to heterogeneity into the meta-regression analyses.

To further investigate the robustness of the pooled results in both groups by sensitivity analyses, a random-effects model was adopted to eliminate one study at a time. None of the studies had a significant influence on the pooled results, indicating that our estimates were robust and reliable (Additional file 1: Figure S1).

### Publication bias

With regards to the non-tumor group, the funnel plot appeared to be asymmetric (Additional file 1: Figure S2A), and statistical significance was observed in Begg’s test (*P* = 0.021), which raised the possibility of publication bias, although no significant publication bias was discovered in Egger’s test (*P* = 0.208). Subsequently, the “trim and fill” method was adopted for the evaluation of the possible impact of publication bias on the pooled effect. In consequence, the symmetric funnel plot was generated through the filling of 10 hypothetical negative articles (Additional file 1: Figure S2B). Typically, the adjusted OR obtained from the pooled analysis incorporating these hypothetical studies was still significant (OR = 5.14, 95% CI = 3.69–7.16, *P* < 0.001). Similarly, for the normal group, both Egger’s test (*P* < 0.001) and the funnel plot revealed the presence of potential publication bias (Additional file 1: Figure S2C), regardless of the absence of statistical significance in Begg’s test (*P* = 0.332). Thereafter, seven hypothetical negative studies were filled through the “trim and fill” approach, but *RASSF1A* promoter methylation was found to be significantly associated with HCC risk in the pooled analyses (OR = 15.71, 95% CI = 7.40–33.36, *P* < 0.001) (Additional file 1: Figure S2D).

### Association of the promoter hypermethylation of *RASSF1A* with HCC-related prognoses

#### Baseline patient characteristics

Data on the promoter methylation of *RASSF1A* were identified within DNA methylation profiles from 380 The Cancer Genome Atlas (TCGA)-derived HCC as well as 50 non-carcinoma samples. Based on UCSC assembly-Dec.2013 (GRCh38/hg38), 11 probes situated at the promoter region of *RASSF1A* were selected (including cg13872831, cg24859722, cg04743654, cg00777121, cg08047457, cg12966367, cg21554552, cg25747192, cg06172942, cg25486143, cg27569446), and they contained the *RASSF1A* gene CpG island A (chr3: 50340373–50341109). In the TCGA cohort, the *RASSF1A* promoter methylation levels within the HCC samples significantly increased compared to those in the adjacent non-carcinoma liver tissues (Additional file 1: Figure S3). According to the probe methylated levels, all samples were classified into the hypomethylated (n = 196) and hypermethylated (n = 184) groups. Among the 380 TCGA-derived HCC samples, 349 had information available on overall survival (OS) and survival status, while 342 had data on disease-free survival (DFS) and recurrence status.

#### *RASSF1A* promoter hypermethylation in the prediction of HCC-related prognoses

**Fig. 5:**
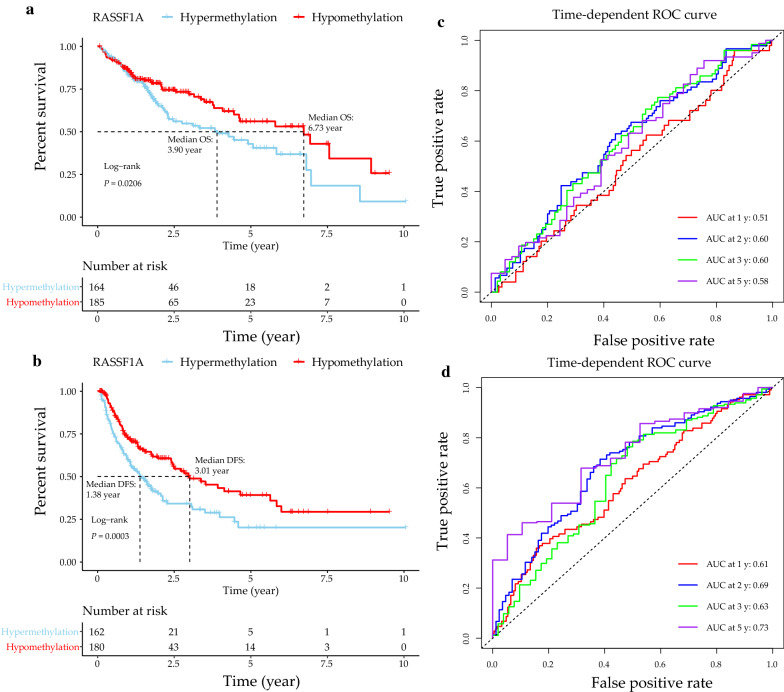
Kaplan-Meier survival and time‐dependent ROC curves of different *RASSF1A* methylation statuses in the TCGA HCC cohort. **a**, **b** Prognostic influences of the promoter hypermethylation of *RASSF1A* on OS and DFS in the TCGA cohort; **c**, **d** Time-dependent ROC curve analysis of the predictive power of *RASSF1A* methylation statuses on OS and DFS

In the Kaplan–Meier survival analysis, HCC cases with *RASSF1A* gene promoter hypermethylation were found to have poorer OS (median OS: 3.90 years vs. 6.73 years; *P* = 0.0206) and DFS (median DFS: 1.38 years vs. 3.01 years; *P* = 0.0003) values than the hypomethylated cases (Fig. 5a, c). Additionally, receiver operating characteristic (ROC) curve analysis was conducted for the determination of the sensitivity and specificity of *RASSF1A* gene promoter hypermethylation in prognosis prediction. The areas under the curve (AUCs) pertaining to *RASSF1A* gene promoter hypermethylation in the prediction of the OS of HCC patients at 1, 2, 3 and 5 years were 0.51, 0.60, 0.60 and 0.58, respectively (Fig. 5b). Meanwhile, the time-dependent AUC values concerning *RASSF1A* gene promoter hypermethylation in the prediction of the OS of HCC patients at 1, 2, 3 and 5 years were 0.61, 0.69, 0.63 and 0.73, separately (Fig. 5d). Accordingly, we inferred that *RASSF1A* gene promoter methylation status exhibited high sensitivity and specificity.

## Discussion

In this study, we found that *RASSF1A* promoter hypermethylation is a promising biomarker for the diagnosis of HCC from tissue and peripheral blood. A number of factors participate in liver carcinogenesis, such as hepatitis virus infection, as well as environmental, genetics and epigenetic alterations [4, 60]. *RASSF1A*, a key tumor suppressor protein, controls cell cycle regulation and cell apoptosis [61, 62]. In 2002, Zhang et al. first reported that the promoter hypermethylation of *RASSF1A* was a major inactivating event in 85% (70/82) of HCC patients [18]. Since then, numerous studies with small sample sizes have demonstrated that the rate of *RASSF1A* promoter methylation is significantly increased within HCC tissues relative to non-carcinoma tissues [36, 44, 59], and the results of association analyses were consistent across studies [26, 35, 57]. Then, Zhao et al. conducted a meta-analysis that involved a total of seven case–control studies, which suggested that the promoter hypermethylation of *RASSF1A* within body fluids was significantly correlated with HCC risk [11]. However, some issues require further clarification, due to which we conducted the present updated study. A large number of studies investigating the association of *RASSF1A* promoter hypermethylation with HCC risk have been published since 2013. Further, the impact of the promoter hypermethylation of *RASSF1A* within cancer tissues on the risk and clinicopathological characteristics of HCC has not been summarized yet, and there was a need for the association of the promoter hypermethylation of *RASSF1A* with HCC prognoses to be analyzed. In our meta-analysis, in which we enrolled 44 articles and 9354 cases, *RASSF1A* promoter hypermethylation showed significant associations with HCC risk within tissues and peripheral blood samples, suggesting that it represents an early event in liver carcinogenesis. Data from the TCGA database indicated that *RASSF1A* gene promoter hypermethylation is significantly correlated with HCC risk. Additionally, two clinicopathological parameters, HBV infection and tumor size, were also found to be associated with *RASSF1A* promoter hypermethylation.

In at least 37 types of cancers, promoter hypermethylation is reportedly directly associated with absent *RASSF1A* gene expression [9, 63]. The *RASSF1A* gene was firstly recognized as a possible RAS-binding molecule in the promotion of apoptosis, due to the presence of an RAS-associated domain within the primary sequence [64]. Dammann et al. demonstrated that the *RASSF1A* gene has a role in tumor suppression, and that its functional loss results in the proliferation of cells and carcinogenesis [62]. Some studies suggest that the *RASSF1A* gene may also be involved in the stabilization of microtubules, regulation of DNA repair, and control of cell cycle and apoptosis [61, 65–67]. The methylation and inactivation of *RASSF1A* exert the most relevant cell protection functions via the inactivation of the Hippo and Wnt signaling pathways, as proven in HCC patients [68–70]. Compared to tumor tissues, *RASSF1A* promoter methylation is not commonly observed in normal tissues. In addition, high *RASSF1A* promoter hypermethylation rates are reported in many cancers, making them potential molecular markers for cancer diagnosis. In the present meta-analysis, the rate of *RASSF1A* promoter methylation within both the HCC tissues and peripheral blood samples apparently increased relative to that within the normal samples, and identical results were reported for non-tumor samples. This indicates that *RASSF1A* promoter hypermethylation may contribute to the entire HCC development process. Furthermore, this study also evaluated the prognostic value of *RASSF1A* promoter hypermethylation within HCC tissue samples. We found that *RASSF1A* promoter hypermethylation was related to poor OS and DFS values. Similar to our results, other studies have also demonstrated that the promoter hypermethylation of *RASSF1A* in peripheral blood has prognostic potential in HCC [53, 55, 71]. Taken together, these results suggest that the detection of *RASSF1A* promoter hypermethylation in tissue and peripheral blood samples may not only serve as a potential diagnostic biomarker for HCC, but also have essential prognostic value in HCC.

Regarding the association of *RASSF1A* promoter hypermethylation with the clinicopathological features of HCC, we discovered that the former is related to HBV infection. Several recent studies have suggested the presence of a relationship between DNA methylation and HCC in association with HBV infection in patients with HCC [72–75]. Some recent studies have suggested that chronic inflammation may be associated with aberrant gene promoter methylation and silencing in ulcerative colitis and gastritis [76, 77]. Moreover, other internal or environmental stimulating factors, including viral infection and hypoxia, may also cause the spread of epigenetic alterations as silent events [9]. It has been demonstrated in certain studies that HBV X protein (HBx) plays an essential role in HBV-related HCC genesis [78, 79]. HBx upregulates the expression of DNA-methyltransferase1 (DNMT1) as well as DNMT3b, thus inducing tumor suppressor gene (TSG) hypermethylation [6, 80]. Additionally, Schagdarsurengin et al. investigated the role of *RASSF1A* during liver carcinogenesis in vitro, they analysed the methylation status of the *RASSF1A* promoter in HBV-positive human hepatocellular carcinoma cell line (Hep3B) and found *RASSF1A* promoter CpG island was hypermethylated [20]. The same result was also revealed by Zhang and his colleagues [18]. They also demonstrated that hypermethylation of *RASSF1A* was detected in Hep3B cells but not in HepG2 cells [18]. The similar results have been yielded in nasopharyngeal carcinoma (NPC) with Epstein–Barr virus (EBV) infection [81]. Lo et al. found that promoter hypermethylation and transcription silencing of RASSF1A were consistently detected in all EBV-positive NPC cell lines [82]. The expression of EBV latent proteins will constitutively activate multiple signaling pathways, enhance genetic instability, induce epigenetic changes, modulate microenvironment and erase host immune response during early stage of cancer development [81].

Nonetheless, a larger number of studies in vitro are warranted to clarify the effect of HBV infection on DNA methylation in the induction of HCC development. Additionally, Okamoto et al. revealed that HBV and HCV infection activates the innate immune response dependent on the natural killer cells to induce DNA methylation, including the *RASSF1A* gene [75]. In this study, only four enrolled studies investigated the association of HCV infection with *RASSF1A* methylation; nevertheless, the results of our pooled analysis were not statistically significant. Future studies should examine the possible biological mechanisms underlying hepatitis virus-caused DNA methylation within the context of HCC. Interestingly, the promoter hypermethylation of *RASSF1A* was significantly related to tumor size, suggesting that the level of *RASSF1A* methylation may reflect tumor load, consistent with previous studies [51, 58, 71]. *RASSF1A*, the TSG associated with cell cycle, inhibits cyclin D1 accumulation and induces cell cycle arrest at the G1 phase [83, 84]. *RASSF1A* promoter hypermethylation also promotes the cell cycle process in those impaired hepatocytes through the escape from arrest at the G1 phase [10]. Nevertheless, statistical significance was not noted in the additional clinicopathological features. This is possibly due to the small sample size and high heterogeneity. Further studies are warranted to examine the association of *RASSF1A* gene promoter hypermethylation with the clinicopathological features of HCC.

Given the moderate to high heterogeneity detected in the relationship of *RASSF1A* gene promoter hypermethylation with HCC risk, this study further conducted meta-regression and subgroup analyses. However, we were unable to identify any factor that significantly contributed to the heterogeneity level in the aforementioned two analyses. Therefore, further studies are needed to examine the effects of those factors. Typically, the clinical and methodological heterogeneities detected across all the enrolled articles represent an essential issue. In this meta-analysis, a large variety of assay methods was used in each study, including a total of seven diverse techniques. Moreover, different thresholds were applied for the assessment of DNA methylation in each study. Non-quantitative methods such as MSP were utilized for the detection of DNA methylation, which precluded the necessity to determine a cut-off point. Previous studies reported different *RASSF1A* hypermethylation rates in HCC using different sets of CpGs [18, 26, 44, 85]. Apart from the selection of the detection technique and thresholds, the determination of the precise genomic positions of those CpG dinucleotides analyzed is also of great importance [86]. With regards to clinical heterogeneity, only 20.5% (9/44) of our enrolled articles mentioned the presence or absence of preoperative treatment [38, 40, 41, 45, 47, 50, 53, 55]. Some studies suggested that both radiotherapy and chemotherapy can alter a patient’s DNA methylation status; therefore, the type of preoperative treatment performed should be clarified [87, 88]. Moreover, four studies investigated the relationship between *RASSF1A* hypermethylation in peripheral blood and the risk of HCC, and the diagnosis of HCC was confirmed by imaging techniques and serum AFP levels, rather than through pathological examination [56–59]. The diagnosis of HCC based on imaging techniques is recommended by guidelines; however, histopathological evaluation remains the gold standard for HCC diagnosis [89, 90]. This is because imaging is not always specific, and there is limited expertise and lack of advanced imaging in many medical centers. Additionally, some studies enrolled in the present study had a retrospective design with a small sample size, which may have led to selection bias [91]. Future studies must examine the effects of the aforementioned factors. Furthermore, normalization of the methods used for the analysis of methylation status, use of uniform definitions, and presence of cooperation among different research groups to obtain a large sample size may be beneficial to studies focusing on the role of methylation marker alterations in cancer.

Reactivation of the TSG that is silenced epigenetically is considered a promising anti-tumor treatment strategy. Over the last few decades, different inhibitors of DNA methylation and natural compounds have been tested in different cancers [92]. In particular, 5-aza-2′-deoxycytidine (Dacogen or Decitabin) and 5-aza-cytidine (Azadine or Vidaza) can lead to *RASSF1A* promoter demethylation and the reactivation of *RASSF1A* expression in diverse types of tumor cells [62, 93–95]. Our study further demonstrated that the promoter hypermethylation of *RASSF1A* was not only a prognostic indicator but also an emerging therapeutic target against HCC.

Nonetheless, several limitations should be noted in this study. First, the funnel plots of both the non-tumor group and normal group were slightly asymmetric, indicating the presence of potential publication bias. However, the results were not significantly changed by the “trim and fill” method, suggesting that the relationship of *RASSF1A* promoter methylation with HCC was meaningful, but not an artifact caused by unpublished negative studies. Second, heterogeneity was present in the current study, which may have been a result of numerous factors. We did not identify any factors that made significant contributions to heterogeneity in the meta-regression and subgroup analyses. Finally, only some of our enrolled studies investigated the relationship of *RASSF1A* promoter methylation with HCC-related prognoses. In this regard, the prognostic role of *RASSF1A* was only investigated among patients from the TCGA HCC cohort. Consequently, a larger number of high-quality studies are warranted to resolve the limitations mentioned above.

## Conclusions

In the present study, the significance of the promoter hypermethylation of *RASSF1A* in HCC diagnoses and prognoses was examined. We found that the rate of *RASSF1A* promoter hypermethylation was increased among HCC patients compared to healthy people and those without HCC. Moreover, *RASSF1A* promoter hypermethylation was significantly related to HBV infection and tumor size, and showed associations with worse prognoses in HCC. Therefore, in addition to its diagnostic value, *RASSF1A* promoter hypermethylation may also be used as a valuable prognostic marker and an emerging target for anti-HCC treatment; further high-quality, well-designed prospective studies are needed to confirm the same.

## Supplementary information

**Additional file 1.** Additional tables and figures.

## Data Availability

All the original data of the current study are available from the corresponding author on reasonable request.

## Abbreviations

RASSF1A: RAS association domain family protein 1A
MSP: Methylation-specific polymerase chain reaction
OR: Odds ratio
CI: Confidence interval
OS: Overall survival
DFS: Disease-free survival
TCGA: The Cancer Genome Atlas
NOS: Newcastle-Ottawa scale
LC: Liver cancer
HCC: Hepatocellular carcinoma
AUC: Area under the curve
ROC: Receiver operating characteristic
HBV: Hepatitis B virus
HBx: Hepatitis B virus X protein
HCV: Hepatitis C virus
AFP: Alpha fetoprotein
