# Supplementary material for: Identification of RASSF1A promoter hypermethylation as a biomarker for hepatocellular carcinoma
Source: Cancer Cell Int. 2020 Nov 10;20:547. doi: 10.1186/s12935-020-01638-5 (PMC7653745; doi:10.1186/s12935-020-01638-5)
Supplement: Supplementary file 1 — Additional file 1. Additional tables and figures. [file 12935_2020_1638_MOESM1_ESM.docx]

**Additional file**

# Additional Figures and Tables

## Additional Tables

**Table S1**: The Newcastle-Ottawa scale (NOS) quality assessment of the enrolled studies.

| Study ID | SELECTION | | | | COMPARABILITY | EXPOSURE | | | Total^a^ |
| --- | --- | --- | --- | --- | --- | --- | --- | --- | --- |
|  | **Adequate definition of cases** | **Representativeness of the cases** | **Selection of controls** | **Definition of controls** | **Comparability of cases and controls^b^** | **Assessment of exposure** | **Same method of assessment for all subjects** | **Non-response rate** |  |
| Yu, 2002 | * | * | — | * | ** | * | * | — | 7 |
| Zhang, 2002 | * | * | — | * | — | * | * | — | 6 |
| Lee, 2003 | * | * | — | * | — | * | * | — | 6 |
| Undraga, 2003 | * | * | — | — | — | * | * | — | 5 |
| Zhong, 2003 | * | * | — | * | ** | * | * | — | 7 |
| Lehmann, 2005 | * | * | — | * | — | * | * | — | 6 |
| Park, 2005 | * | * | — | * | ** | * | * | — | 7 |
| Yeo, 2005 | * | * | — | * | — | * | * | — | 6 |
| Calvisi, 2006 | * | * | — | * | ** | * | * | — | 7 |
| Gioia, 2006 | * | * | — | * | — | * | * | — | 6 |
| OH, 2007 | * | * | — | * | ** | * | * | — | 7 |
| Zhang, 2007 | * | * | * | * | ** | * | * | — | 8 |
| Zhang, 2007 | * | * | — | * | ** | * | * | — | 7 |
| Chan, 2008 | * | * | — | * | ** | * | * | — | 7 |
| Chang, 2008 | * | * | — | * | — | * | * | — | 6 |
| Nishida, 2008 | * | * | — | * | ** | * | * | — | 7 |
| Su, 2008 | * | * | — | * | ** | * | * | — | 7 |
| Lou, 2009 | * | * | — | * | ** | * | * | — | 7 |
| Hu, 2010 | * | * | — | * | — | * | * | — | 6 |
| Formeister, 2010 | * | * | — | * | ** | * | * | — | 7 |
| Feng, 2010 | * | * | — | * | — | * | * | — | 6 |
| Saelee, 2010 | * | * | — | * | — | * | * | — | 6 |
| Hua, 2011 | * | * | — | * | ** | * | * | — | 7 |
| Um, 2011 | * | * | — | * | — | * | * | — | 6 |
| Feng, 2012 | * | * | — | * | ** | * | * | — | 7 |
| Li, 2012 | N.A. | N.A. | N.A. | N.A. | N.A. | N.A. | N.A. | N.A. | N.A. |
| Mohamed, 2012 | * | * | — | * | ** | * | * | — | 7 |
| Xu, 2013 | * | * | — | * | ** | * | * | — | 7 |
| Zhang, 2013 | * | * | — | * | ** | * | * | — | 7 |
| Michailidi, 2014 | * | * | — | * | — | * | * | — | 6 |
| Zekri, 2014 | * | * | — | * | ** | * | * | — | 7 |
| Feng, 2015 | * | * | — | * | ** | * | * | — | 7 |
| Huang, 2015 | * | * | — | * | — | * | * | — | 6 |
| Lin, 2015 | N.A. | N.A. | N.A. | N.A. | N.A. | N.A. | N.A. | N.A. | N.A. |
| Qu, 2015 | * | * | — | * | — | * | * | — | 6 |
| Kanekiyo, 2015 | N.A. | N.A. | N.A. | N.A. | N.A. | N.A. | N.A. | N.A. | N.A. |
| Villanueva, 2015 | * | * | — | * | — | * | * | — | 6 |
| Dong, 2015 | * | * | — | * | ** | * | * | — | 7 |
| Araújo, 2016 | * | * | — | * | — | * | * | — | 6 |
| Liu, 2017 | * | * | — | * | — | * | * | — | 6 |
| Mansour, 2017 | * | * | — | * | — | * | * | — | 6 |
| Wu, 2017 | * | * | * | * | ** | * | * | — | 8 |
| Bendary, 2019 | * | * | — | * | ** | * | * | — | 7 |
| Pasha, 2019 | * | * | — | * | ** | * | * | — | 7 |

— indicates zero score, * indicates one score, ** indicates two scores. N.A. not available

^a^ Each study could be awarded a maximum of nine stars: a maximum of two stars for the item regarding comparability and a maximum of one star for other 7 items.

^b^ A maximum of two stars could be awarded for this item. If the cases and controls in a study were matched for age, one score was awarded. If the cases and controls in a study were matched for any other factors, an additional score was awarded. If the cases and controls in a study derived from the same population (eg. tumors and adjacent non-tumorous tissue), two scores were awarded.

**Table S2**: Meta-regression indicating the impact of variables on the effect size.

| Variables | *P* for covariates | REML estimate of between-study variance (τ^2^) | Residual variance due to heterogeneity (I^2^) | Proportion of between-study variance explained |
| --- | --- | --- | --- | --- |
|  |  |  |  |  |
| Non-tumor | | | | |
| Sample type | 0.833 | 0.545 | 62.62% | -9.70% |
| Detection method | 0.429 | 0.506 | 64.68% | -1.85% |
| Sample size | 0.768 | 0.526 | 65.21% | -5.69% |
| Normal | | | | |
| Sample type | 0.804 | 3.210 | 78.55% | -5.92% |
| Detection method | 0.426 | 3.226 | 79.93% | -6.47% |
| Sample size | 0.178 | 2.784 | 73.58% | 8.14% |

Abbreviations: REML, restricted maximum likelihood

## Additional Figures


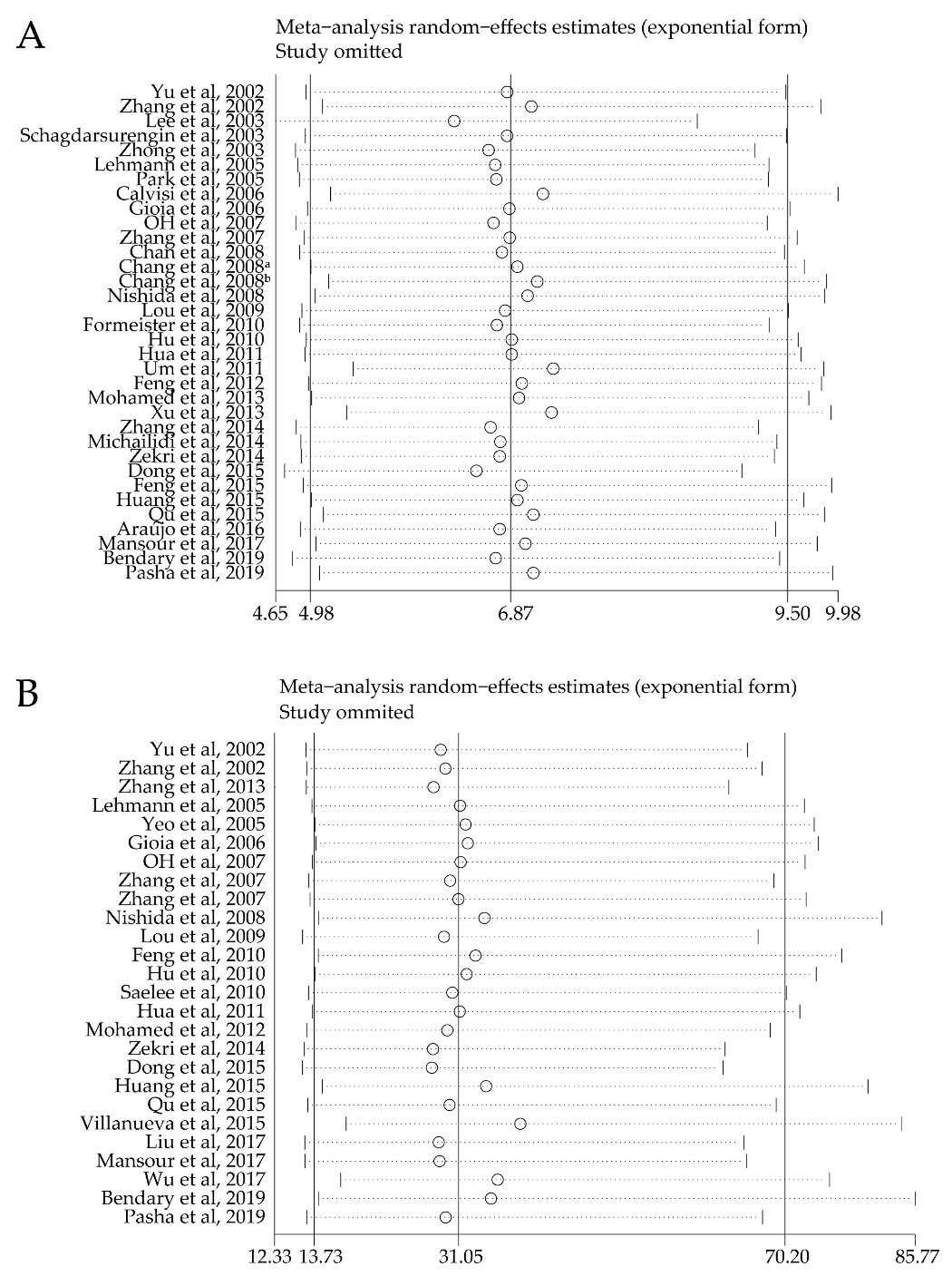


**Supplementary Figure S1**. Sensitivity analysis of the pooled results. (A) Sensitivity analysis of RASSF1A promoter hypermethylation on HCC in non-tumor group; (B) Sensitivity analysis of RASSF1A promoter hypermethylation on HCC in normal group.


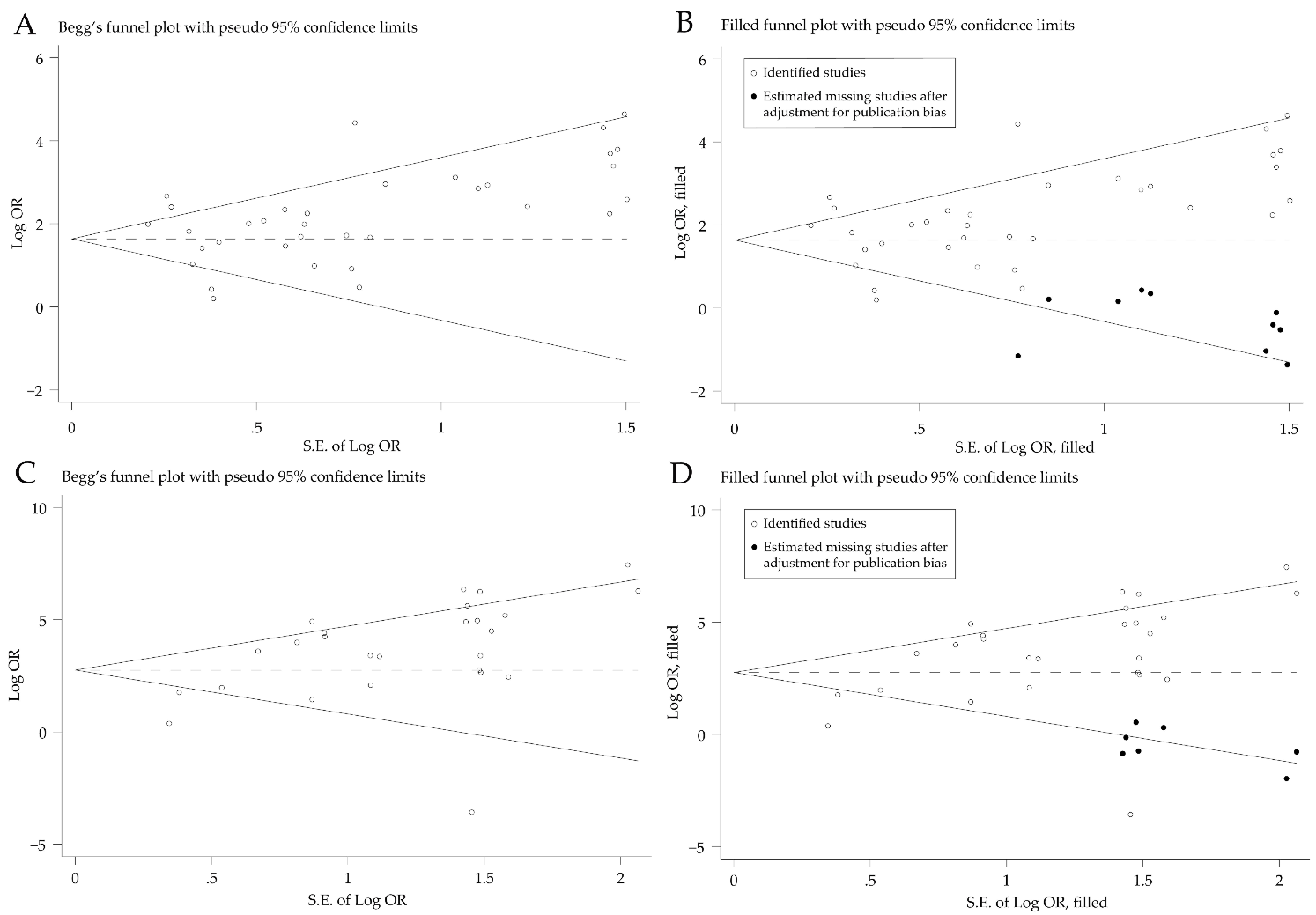
**Additional Figure S2**: Publication bias tests of the pooled results. (A and C) Begg’s funnel plots for publication bias of *RASSF1A* promoter hypermethylation on HCC without trim and fill in non-tumor group and normal group. (B and D) Begg’s funnel plots for publication bias of *RASSF1A* promoter hypermethylation on HCC group with trim and fill in non-tumor group and normal group.

**Additional Figure S3**: TCGA data showing the expression profiles of the *RASSF1A* methylation statuses in tumour tissue (n = 380) vs normal liver (n = 50).
